# Phosphorylation of ASPP2 by RAS/MAPK Pathway Is Critical for Its Full Pro-Apoptotic Function

**DOI:** 10.1371/journal.pone.0082022

**Published:** 2013-12-02

**Authors:** Nadia Godin-Heymann, Yihua Wang, Elizabeth Slee, Xin Lu

**Affiliations:** Ludwig Institute for Cancer Research Ltd., Nuffield Department of Clinical Medicine, University of Oxford, Oxford, United Kingdom,; Peking University Health Science Center, China

## Abstract

We reported recently that apoptosis-stimulating protein of p53 (ASPP) 2, an activator of p53, co-operates with oncogenic RAS to enhance the transcription and apoptotic function of p53. However, the detailed mechanism remains unknown. Here we show that ASPP2 is a novel substrate of mitogen-activated protein kinase (MAPK). Phosphorylation of ASPP2 by MAPK is required for RAS-induced increased binding to p53 and increased transactivation of pro-apoptotic genes. In contrast, an ASPP2 phosphorylation mutant exhibits reduced p53 binding and fails to enhance transactivation and apoptosis. Thus phosphorylation of ASPP2 by RAS/MAPK pathway provides a novel link between RAS and p53 in regulating apoptosis.

## Introduction

p53 is the most commonly mutated tumour suppressor protein thus far identified. Although p53 is mutated in more than 50% of tumours, its mutation rate varies significantly between different types of human cancer, with a particularly high incidence in colorectal and pancreatic tumours. Interestingly, the mediator of signal transduction *RAS* is also commonly mutated in these particular tumour types. It remains unclear why there exists such a tight association between the *p53* and *RAS* mutation status [1]. We reported recently that apoptosis-stimulating protein of p53 (ASPP) 2 co-operates with oncogenic RAS to enhance the transcription and apoptotic function of p53 in cancer cells [2]. This may be achieved via the ability of active RAS to induce ASPP2, thereby promoting ASPP2’s interaction with p53 and enhancing the activity of p53. However, the detailed mechanism underlying this observation remains to be elucidated. 

 Activated RAS promotes the protein kinase activity of RAF, which phosphorylates and activates MEK (also known as MAPKK). MEK phosphorylates and activates a mitogen-activated protein kinase (MAPK/ERK), a serine/threonine-selective protein kinase. The MAPK enzymes require a specific phosphorylation sequence where a serine or threonine is followed by proline (S/TP) [3]. It was shown that endogenous RAS is necessary for the full apoptotic activity of ASPP2, which suggests that RAS signalling may modify ASPP2, potentially via a phosphorylation event. Phosphorylation by RAS/MAPK modulates the activation of most of their substrates and in some cases the phosphorylation mediates changes in subcellular localisation [4].

ASPP2 belongs to an evolutionarily conserved ASPP family of proteins, alongside ASPP1 and iASPP. All three contain signature sequences in their C-termini; ankyrin repeats, SH3 domain and proline rich sequences [5]. ASPP2 binds to RAS through its N-terminus [2,6]. The functions of ASPP2 are potentially controlled by its binding partners and localisation. When ASPP2 locates at the cell-cell junctions, it binds and co-localises with PAR3 via its N-terminus to maintain the integrity of cell polarity and adherence junction [7,8], whereas in the cytosol/nucleus, ASPP2 enhances p53-induced apoptosis in cancer cells [9]. It also binds ATG5 and inhibits RAS-induced autophagy, independently of p53 [10]. Thus it is important to find the molecular event that controls the localisation of ASPP2. 

 Here we show that ASPP2 is a novel substrate of RAS/MAPK. Phosphorylation of ASPP2 by MAPK is required for the RAS-induced translocation of ASPP2, which results in the increased binding to p53. Consequently, the pro-apoptotic activity of ASPP2 is increased by the RAS/Raf/MAPK signalling cascade as ASPP2 phosphorylation mutant fails to do so. Thus phosphorylation of ASPP2 by RAS/MAPK pathway provides a novel link between RAS and p53 in regulating apoptosis.

## Results

### ASPP2 is a novel substrate of MAPK

**Figure 1:**
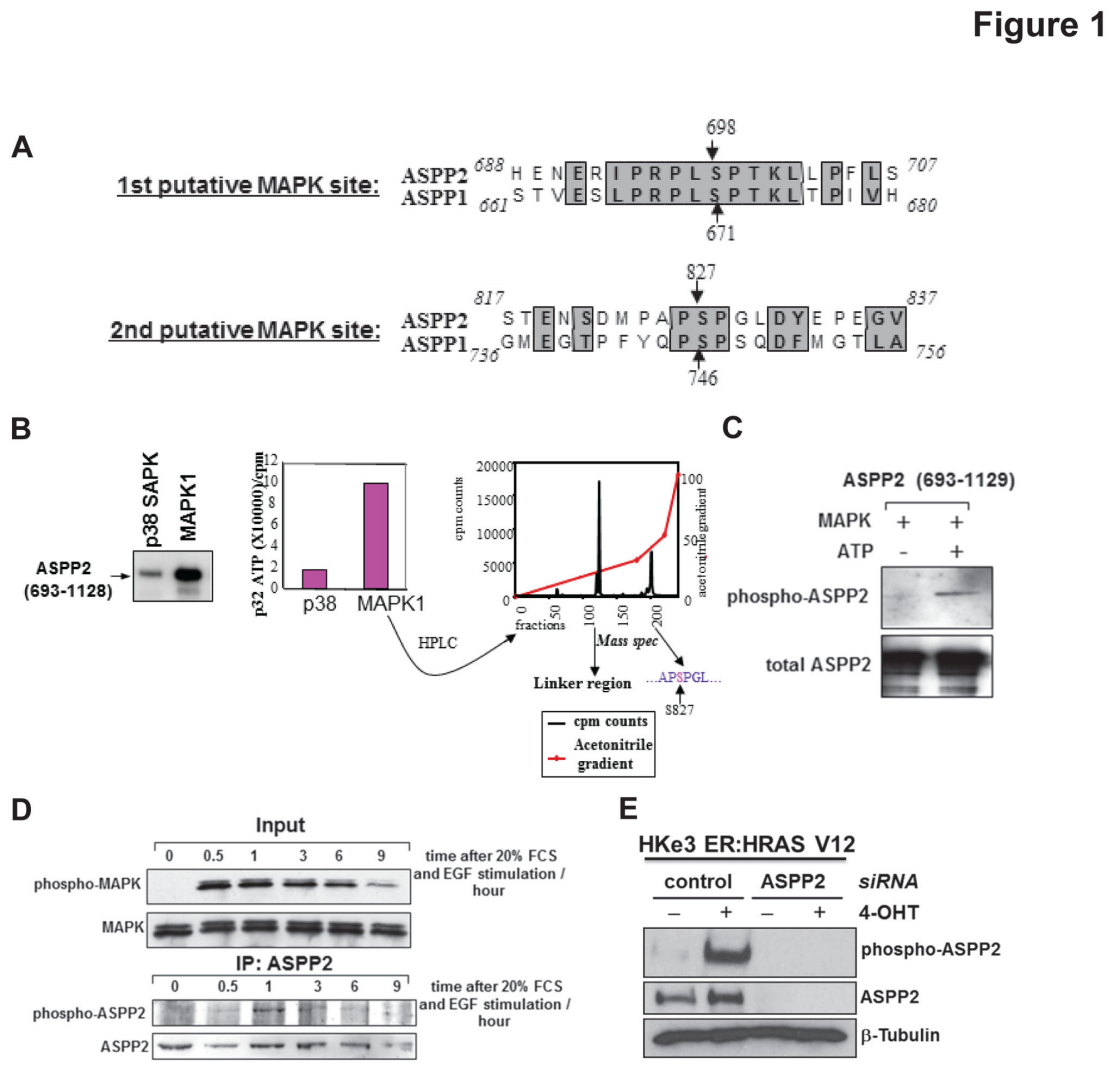
MAPK phosphorylates ASPP2. (**A**) ASPP1 and ASPP2 have two conserved putative MAPK2 phosphorylation sites in their C-terminus. (**B**) The C-terminus fragment of ASPP2 is phosphorylated *in* *vitro* by MAPK1 (left panel). The intensity of phosphorylation is quantified (middle panel). The MAPK1 phosphorylated ASPP2 fragment was digested with trypsin and chromatographed and the radioactive peptides were measured by mass spectrometry (right panel). The first peak represents the GST linker region whereas the second presented a region of equal mass to the fragment containing serine 827. (**C**) An *in* *vitro* phosphorylation assay was performed on the ASPP2 C-terminus fragment with recombinant MAPK1 and non-radioactive ATP. The phosphorylation status of ASPP2 was assessed using the purified NGH.S4 phospho-specific ASPP2 antibody (upper panel). Total ASPP2 is shown in the lower panel. (**D**) Saos2 cells were starved then stimulated with serum and EGF. At the indicated times the cells were harvested and either blotted for phospho/total MAPK (upper panel) or immunoprecipitated for total ASPP2 and blotted with NGH.S4 phospho-ASPP2 antibody. (**E**) Total cell lysates from HKe3 ER:HRASV12 cells treated with or without 4-OHT were transfected with control siRNA or siRNA against ASPP2. ASPP2 phosphorylation was detected with ES1 phospho-ASPP2 antibody and total ASPP2.

 It has recently been shown that oncogenic RAS can enhance the apoptotic function of p53 via ASPP1 and ASPP2. Mechanistically ASPP1 and ASPP2 bind RAS-GTP and potentiates RAS signalling to enhance p53 mediated apoptosis [2]. As RAS is upstream of several signalling cascades [13], we queried whether the activity of ASPP2 is regulated by the activation of a RAS-mediated signalling pathway. One of the most studied downstream pathways of RAS signalling is the Raf-MAPK pathway. Interestingly, we observed two conserved putative MAPK phosphorylation sites in ASPP1 and ASPP2. The ASPP1 sites are at residues 671 and 746, and the ASPP2 sites are at residues 698 and 827 (Figure 1A). We thus tested whether RAS activation may regulate ASPP2 phosphorylation. An *in vitro* phophorylation assay was performed with a purified C-terminus fragment of ASPP2 (693-1128) containing both MAPK putative phosphorylation sites. When compared to p38 SAPK, MAPK1 was clearly able to phosphorylate the ASPP2 fragment *in vitro* (Figure 1B, left and middle panels). As shown in Figure S1, histone 2B phosphorylated by p38 SAPK had high levels of incorporated ^32^P, suggesting that p38 SAPK was active; while under the same conditions, ASPP2 (693-1128) fragment phosphorylated by p38 SAPK had very low levels of incorporated ^32^P, indicating that p38 SAPK is not an efficient kinase for ASPP2 phosphorylation. The phosphorylated ASPP2 fragment by MAPK1 was digested by trypsin and fractioned on a high performance liquid chromatography (HPLC). Each eluted fraction was measured for its radioactivity content (Figure 1B, right panel). The fractions representing these radioactive peaks were analysed by mass spectrometry. Of the two radioactive peaks, one represented the linker region between the GST and our ASPP2 fragment and the other corresponded to a fragment of the same mass as that containing the second putative phosphorylation site, serine 827. Hence ASPP2 can be phosphorylated at serine 827 by MAPK1 *in vitro.*

 A synthetic peptide encoding amino acids 824-832, with a phosphoserine at residue 827, was used to raise antibodies. A polyclonal antibody NGH.S4 was purified by affinity column purification. To test the efficacy of the purified phospho-specific antibody, a non-radioactive *in vitro* phosphorylation assay was performed on the purified GST-ASPP2 fragment (693-1128) with recombinant MAPK1. Figure 1C shows that the phospho-specific antibody is specific for the ASPP2 fragment phosphorylated *in vitro* by MAPK. To test whether endogenous ASPP2 could be phosphorylated in cells, Saos2 cells were grown in low serum for 50 hours to remove all background stimulation of RAS, after which the cells were stimulated with EGF and 20% fetal calf serum (FCS). Phosphorylated endogenous ASPP2 was detected by the phospho-specific antibody 30 minutes after RAS stimulation (Figure 1D). ASPP2 phosphorylation was rapid and transient as 3 hours after EGF stimulation phosphorylated ASPP2 was barely detectable. Moreover, with another different phospho-ASPP2 antibody, ES1, ASPP2 phosphorylation was also observed in a human colon cancer cell line HKe3 ER:HRASV12 cells, in which RAS activation is induced upon the addition of 4-hydroxytamoxifen (4-OHT) [2,10,11] (Figure 1E). The phospho-specific antibody for ASPP2 is specific as knockdown of ASPP2 resulted in a lack of detection of phospho-ASPP2. All these demonstrate that ASPP2 is a novel substrate of MAPK and Ser827 of ASPP2 can be phosphorylated by RAS/MAPK pathway.

### Raf/MAPK Pathway Activates the Pro-Apoptotic Function of ASPP2

**Figure 2:**
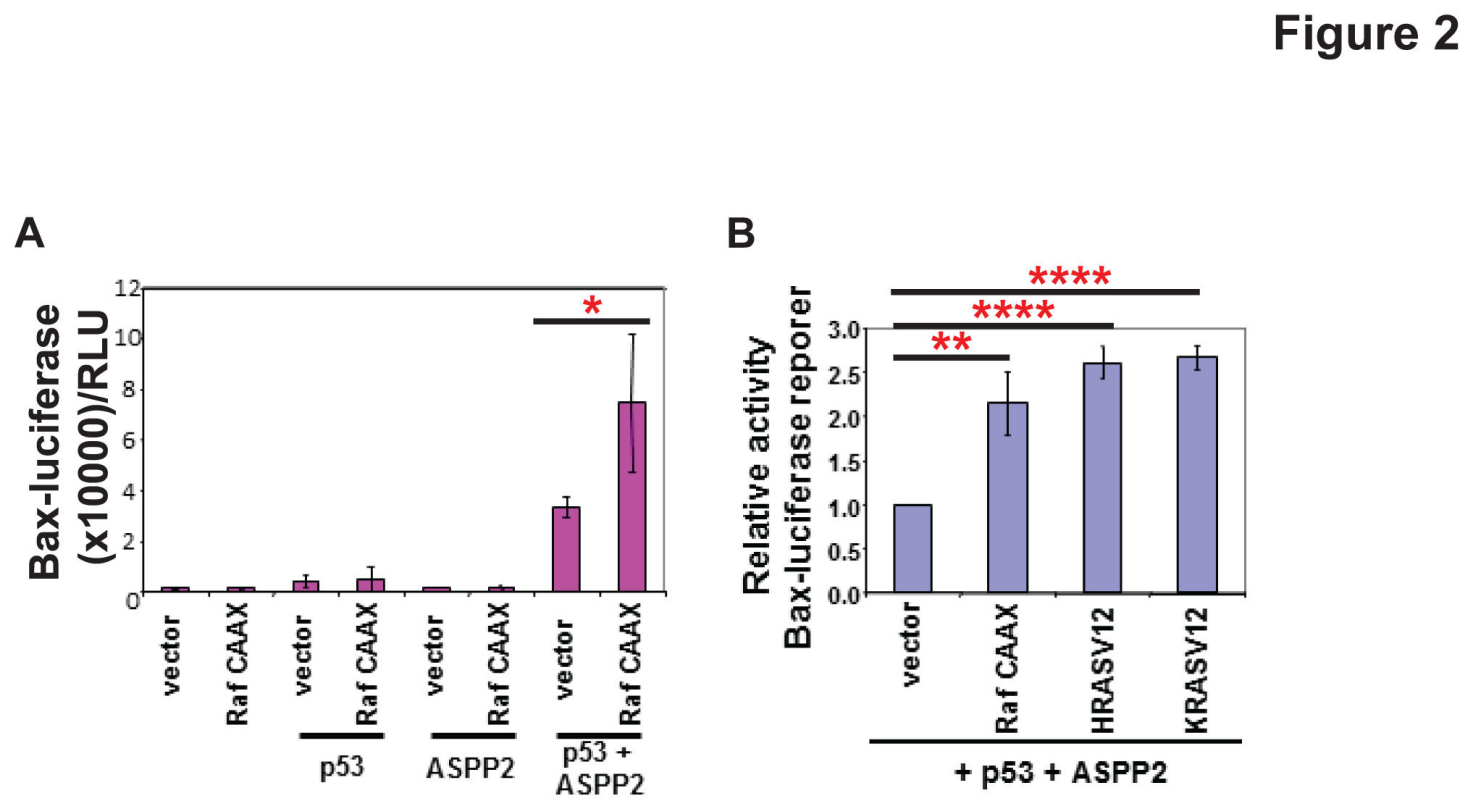
Activated Raf enhances the transactivation activity of ASPP2 and p53 to the same extent as activated RAS. (**A**) Saos2 cells were transfected as indicated with a Bax-luciferase reporter and the luciferase activity shown. * *P*=0.05 (**B**) The value of ASPP2+p53 was taken as 1.0 to reflect the fold increase of ASPP2 and p53 in the presence of activated Raf and mutant RAS. ** *P*=0.0055; **** *P*=0.0001.

 One of the most studied downstream pathways of RAS signalling is the Raf/MAPK pathway [13]. Knowing ASPP2 is a substrate of MAPK, we thus tested whether activation of Raf/MAPK pathway is sufficient to regulate ASPP2 activity using a mutant form of Raf (Raf CAAX), which is constitutively present at the plasma membrane, so the Raf pathway is constitutively active [14]. The impact of co-expression of Raf CAAX with p53 and ASPP2 was tested by analysing the transcriptional activity of p53 on the pro-apoptotic Bax reporter. Raf CAAX increases Bax-luciferase levels by 2.5 fold over the baseline of p53 and ASPP2 alone (*P*=0.05). This effect is likely to be mediated by ASPP2 as Raf CAAX had little effect on p53 in its absence (Figure 2A).

 The effect of Raf CAAX on ASPP2 and p53 was compared to that of HRAS V12 or KRAS V12. All three activate ASPP2 and p53 transactivation activity to a similar extent, namely 2.5 fold (Figure 2B). This suggests that the effect of activated RAS on ASPP2 and p53 is mediated via the downstream Raf /MAPK pathway.

### ASPP2 phosphorylation by MAPK is necessary for ASPP2 full pro-apoptotic activity

**Figure 3:**
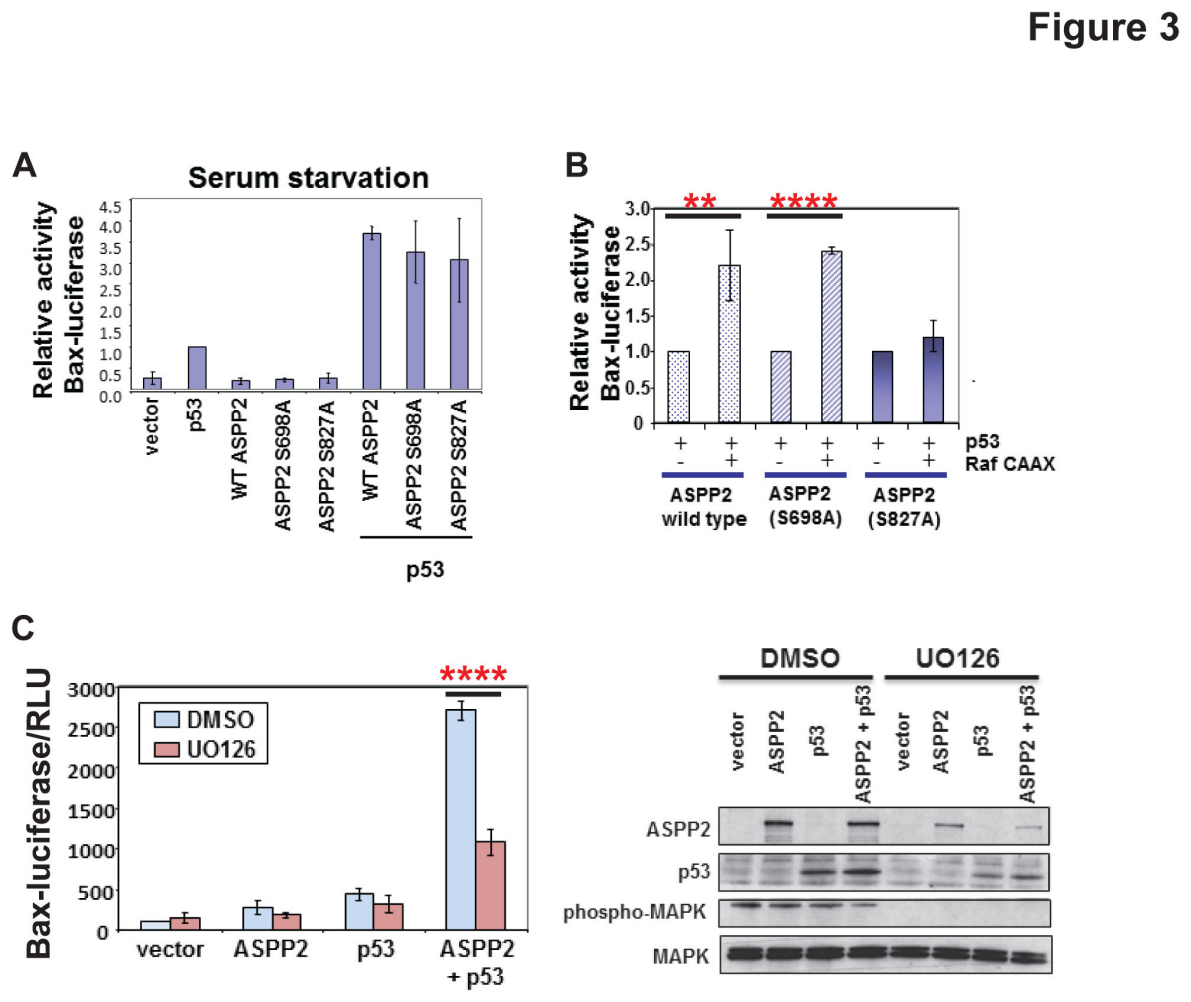
Phosphorylation of ASPP2 by the Raf/MAPK pathway enhances p53-mediated transactivation. (**A**) Saos2 cells were transfected with p53 and either wild-type or mutant ASPP2 as indicated together with a Bax-luciferase reporter. Luciferase activity is shown following harvesting of cells. Values are Relative Light Units (RLU). (**B**) Saos2 cells were transfected with p53, constitutively active Raf CAAX and either wild-type or mutant ASPP2 as indicated together with a Bax-luciferase reporter. ** *P*=0.013; **** *P*=0.0001 (**C**) Saos2 cells were transfected with a Bax-luciferase reporter, ASPP2 and p53 and treated with 20 µM UO126 or DMSO for 20 hours. Luciferase activity is shown in the left panel and protein expression was verified by Western Blot (right panel). **** *P*=0.0001.

 To assess the effect of MAPK phosphorylation on ASPP2 activity, alanine substitution mutants of the two putative MAPK phosphorylation sites were constructed. In conditions where the cells were starved of serum, these mutants had identical activity to wild-type ASPP2 in their ability to enhance the transactivation function of p53 (Figure 3A). However, whereas activated Raf CAAX was able to stimulate wild-type ASPP2 and ASPP2 (S698A) by 2.5 fold, it was unable to increase the activity of mutant ASPP2 (S827A) (Figure 3B). These results suggest that MAPK phosphorylation of ASPP2 Ser827 is necessary for Raf CAAX to stimulate the full transcriptional activity of p53 via ASPP2. 

 To determine the effect of MAPK phosphorylation on endogenous ASPP2 activity, cells were treated with a MAPK inhibitor, UO126. Inhibiting MAPK resulted in a significant decrease in the ability of ASPP2 to enhance the transcriptional activity of p53 compared to the control cells (Figure 3C), further validating the functional role of ASPP2 phosphorylation by MAPK. Moreover, another MAPK inhibitor, PD 98059, also inhibited ASPP2 function to a similar extent (Figure S2). Interestingly, the endogenous ASPP2 protein level was slightly decreased following RAS/MAPK pathway inhibition by UO126 treatment (Figure 3C, right panel) while RAS/MAPK pathway activation resulted in the increased ASPP2 expression (Figure 1E; Figure S3C). The mRNA level of ASPP2 was not affected upon RAS/MAPK pathway activation (Figure S3A), indicating that the regulation is not at the transcriptional level. Thus we investigated whether RAS/MAPK pathway activation could result in the increased protein stability of ASPP2. The ASPP2 protein levels in the presence or absence of oncogenic RAS were measured after cycloheximide (CHX) addition (Figure S3B and C). ASPP2 was a bit more stable when oncogenic RAS is induced in HKe3 ER:HRASV12 cells (Figure S3B) or co-transfected in Saos2 cells (Figure S3C): ASPP2 levels were considerably decreased without oncogenic RAS, while in the presence of HRAS V12, ASPP2 protein levels remained high after CHX treatment. These data indicate that ASPP2 phosphorylation by RAS/MAPK is necessary for ASPP2 full pro-apoptotic activity and this may be mediated by the stabilization of ASPP2 protein.

### Ser827 phosphorylation is required for RAS-induced translocation of ASPP2

**Figure 4:**
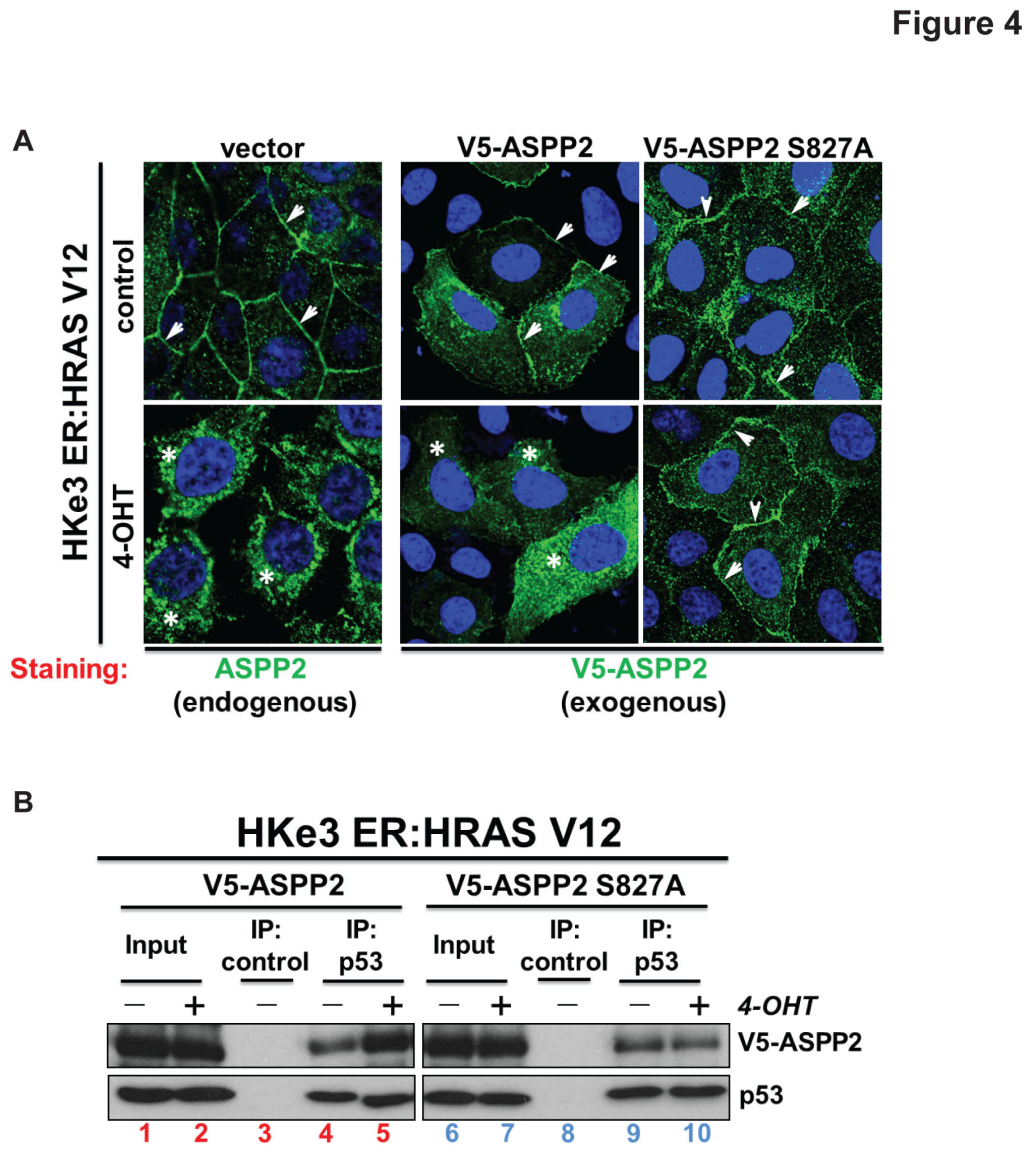
Wild-type ASPP2, but not mutant ASPP2 (S827A), translocates to the cytosol and nucleus upon oncogenic RAS activation and this results in an increased interaction with p53. (**A**) RAS activation induces cytoplasmic and nuclear translocation of wild-type ASPP2 but not ASPP2 (S827A) in HKe3 ER:HRAS12 cells as detected by immunofluorescence. Arrows indicate cell membrane and stars indicate cytosol. (**B**) RAS activation enhances the binding of wild-type ASPP2 but not ASPP2 (S827A) to p53. Total cell lysates from HKe3 ER:HRASV12 cells treated with or without 4-OHT were immunoprecipitated with an anti-p53 antibody or control IgG as indicated.

 It has recently been shown that activation of RAS results in ASPP2 translocation from the plasma membrane to the cytosol and nucleus [2]. We therefore tested whether MAPK phosphorylation at Ser827 could affect the cellular localization of ASPP2. Interestingly, we observed that in contrast to wild type ASPP2, ASPP2 (S827A) remains at the plasma membrane following RAS activation by 4-OHT in the HKe3 ER:HRASV12 system (Figure 4A). This suggests that ASPP2 binding to RAS at the plasma membrane occurs prior to MAPK phosphorylation of ASPP2 and that Ser 827 phosphorylation is required for RAS-induced translocation of ASPP2 to the cytosol.

 As the translocation of wild-type ASPP2 from the plasma membrane to the cytosol and nucleus results in increased binding to p53 [2], we tested whether the p53 binding ability of ASPP2 phosphorylation mutant would be influenced by RAS activation. Indeed, upon activation of RAS by 4-OHT in HKe3 ER:HRASV12 cells, we observed an increase in the amount of p53 co-immunoprecipitated with transfected V5-tagged wild-type ASPP2 (Figure 4B, compare lanes 4 and 5). Under the same conditions, the amount of p53 in complex with transfected V5-ASPP2 (S827A) is not affected by RAS activation (Figure 4B, compare lanes 9 to 10). Together these data illustrate that phosphorylation of ASPP2 at Ser827 by MAPK is necessary for its ASPP2 to fully enhance p53’s pro-apoptotic activity. 

## Discussion

 ASPP2 is a haploinsufficient tumor suppressor [15] [16] and it can cooperate with p53 to suppress tumour growth *in vivo* [15]. In human cancer, ASPP2 expression is down-regulated by DNA methylation [9,17-19]. Importantly, in diffuse large B cell lymphomas, reduced ASPP2 expression associates with poor prognosis [20]. ASPP2 expression is also down-regulated in both invasive and metastatic cells compared with normal breast epithelium [21]. These findings established ASPP2 as a tumor suppressor and an activator of p53 family. ASPP2 is involved in both senescence in fibroblasts and apoptosis in cancer cells [2,10]. ASPP2 acts at several steps in promoting senescence in fibroblasts. It can do so in a RAS-dependent, p53-independent manner, through its ability to bind ATG5 and to inhibit RAS-induced autophagy [10]. Additionally, it can bind active RAS directly leading to enhanced activation of c-Raf/MAPK-mediated senescence [6].

**Figure 5:**
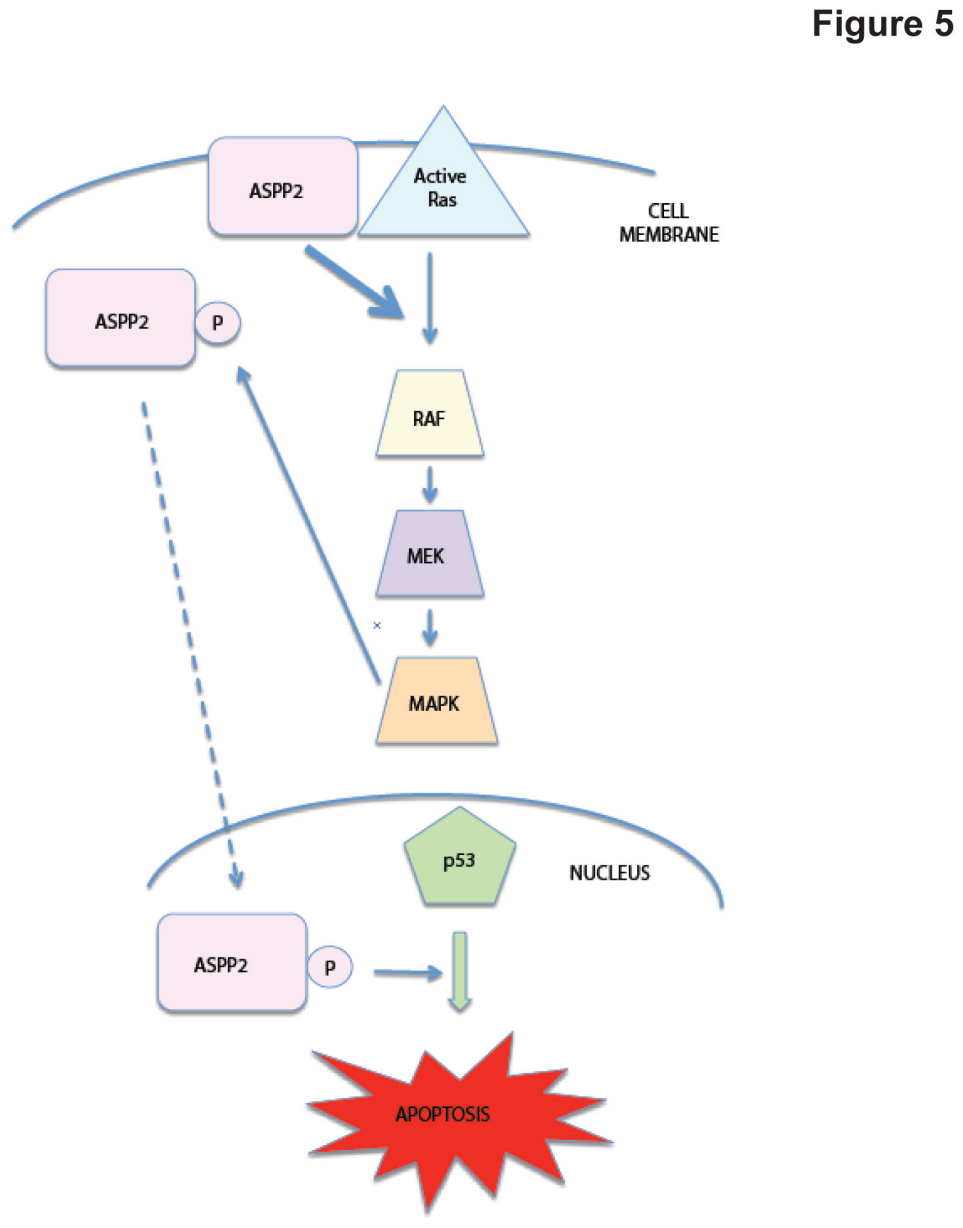
Diagram summarizes the inter-regulation between ASPP2 and RAS. ASPP2 binds active RAS at the plasma membrane, thereby increasing RAS signaling to its downstream pathway effectors Raf/MAPK. Activated MAPK phosphorylates ASPP2 which can then relocate to the nucleus and activate p53 pro-apoptotic signaling.

 We and others have recently shown that ASPP2 can potentiate RAS signaling by binding directly via the ASPP2 N-terminus [2,6]. Moreover, the RAS-ASPP interaction enhances the transcription function of p53 in cancer cells [2]. Until now, it has been unclear how RAS could affect ASPP2 to enhance p53 function. We show here that ASPP2 is phosphorylated by the RAS/Raf/MAPK pathway and that this phosphorylation leads to its increased translocation to the cytosol/nucleus and increased binding to p53, providing an explanation of how RAS can activate p53 pro-apoptotic functions (Figure 5). Additionally, RAS/Raf/MAPK pathway activation stabilizes ASPP2 protein, although the underlying mechanism remains to be investigated.

 Thus ASPP2 is both upstream of RAS, by binding active RAS and enhancing its downstream pathway activity, as well as downstream of the RAS/RAF/MAPK pathway. It is likely that this possible feedback loop leads to an amplified pro-apoptotic signal. In a scenario where RAS is mutated and thereby constitutively active, it binds ASPP2, resulting in increased RAS/MAPK signaling. This in turn activates ASPP2 via MAPK phosphorylation which will translocate and activate p53 to promote apoptosis. ASPP2 will also continue to bind active RAS, thereby propagating the pro-apoptotic signal from RAS to p53. If RAS is only temporarily active, as happens in natural growth conditions, one could hypothesize that ASPP2 would bind active RAS, potentiate its downstream pathways, and MAPK-mediated phosphorylation of ASPP2 would lead to increased binding to p53. However, once RAS reverts to its inactive state, it would be unable to bind to ASPP2 and would therefore be unable to amplify the RAS signal to p53. Only in conditions where RAS is constitutively active would the ASPP2 feedback loop reach a threshold of signaling high enough to result in p53-dependent apoptosis. The ability of oncogenic RAS to stimulate apoptosis allows the cell to have a fail-proof mechanism: mutated RAS signals to p53 via ASPP2 to induce apoptosis instead of uncontrolled proliferation. However, under normal conditions, growth factors or growth receptors would only activate RAS for short periods of time, preventing the amplification of ASPP2 signaling to p53 via the feedback loop, thereby eliminating the apoptotic stimulus. The novel RAS/Raf/MAPK/ASPP2 pathway is thus involved in an important feedback loop between RAS and p53, and is an effective way for mutant RAS to induce apoptosis in cancer cells with wild-type p53. 

## Materials and Methods

### Cell culture, plasmids and siRNA

 Saos2 cells were obtained from the American Type Culture Collection (Manassas, VA). HKe3 ER:HRASV12 cells were kindly provided by Dr Julian Downward [2,10,11]. Cells were cultured in DMEM supplemented with 10% fetal bovine serum (FBS) and penicillin streptomycin (Gibco-BRL, Invitrogen Ltd, Paisley, UK). For growth factor stimulation, cells were serum starved for indicated time, followed by stimulation with 20% serum plus epidermal growth factor (EGF), EGF alone or insulin (Sigma, Dorset, UK) as indicated.

 Full-length ASPP2 and ASPP2 (693-1128) was tagged with the V5-epitope. HRASV12 and KRASV12 were tagged with the haemagglutinin (HA) epitope. All expression plasmids used in this study were driven by the cytomegalovirus immediate-early promoter, with the exception of the RAS and Raf CAAX plasmids which were driven by the EF1a promoter. The ASPP2 mutants were constructed using the QuickChange Site-Directed Mutagenesis Kit (Stratagene). 

 siRNA oligos against ASPP2 were purchased from Dharmacon. Sequences are available from Dharmacon or upon request. We used siGENOME RISC-Free siRNA (Dharmacon, Lafayette, CO, United States) as a negative control. Cells were transfected with the indicated siRNA oligos at a final concentration of 35nM using Dharmafect 1 reagent (Dharmacon, Lafayette, CO, United States), according to the manufacturer's instructions.

### Generation of Phosphor-ASPP2 Antibody

 Using the peptide CPAPSpPGLDY (representing residues 824-832) with the serine phosphorylated as an immunogen, a rabbit polyclonal antibody NGH.S4 which specifically recognizes phosphorylated ASPP2 at amino acid 827 was raised. An affinity column was made by cross-linking the phospho-peptide to epoxy-sepharose-6B (Amersham Pharmacia Biotech) according to the manufacturer’s instructions. Serum from the final bleed was clarified by centrifugation and filtration through a 0.45μm filter and was supplemented with 1X TTBS (0.5 M NaCl, 20 mM Tris [pH 8.0], 0.1% Tween-20). This was passed over the affinity column and washed with TTBS until the flow-through had an OD_280nm_ <0.01. The antibody was then eluted with 0.2M glycine (pH 2.8) and neutralized with Tris-HCl (pH 8.0). A second phospho-specific antibody ES1 was raised against the peptide SDMPAP**S**[P]PGLDYE where **S**[P] is the phosphorylated serine 827, and was conjugated to KLH. The serum was double affinity purified against the non-phosphorylated peptide SDMPAPSPGLDYE.

### Transactivation Assay

 Saos2 cells (7 x 10^5^) were plated 24 hours prior to transfection in 6-cm-diameter dishes. All transactivation assays contained 1 μg of reporter plasmid. 50 ng of p53, 4 μg of ASPP2, and 1.5 μg of HRASV12 or KRASV12 or Raf CAAX expression plasmids were used as indicated. Cells were lysed in reporter lysis buffer 16 to 24 hours after transfection and assayed using the luciferase assay kit (Promega). The fold increase of p53 and ASPP by HRASV12/KRASV12/Raf CAAX was determined by the activity of p53 and ASPP in combination with RASV12/Raf CAAX divided by the activity of p53 and ASPP alone. 

### Western blot analysis

 For Western blotting, cells were lysed in either NP40 lysis buffer (1% Nonidet P40, 50 mM Tris [pH 8.0], 150 mM NaCl, and 1 mM EDTA), RIPA buffer (150 mM NaCl, 1% Nonidet P40, 0.5% sodium deoxycholate, 0.1% sodium dodecyl sulfate, 50 mM Tris-HCl [pH 8.0]), Urea solution (8 M Urea, 1 M Thiourea, 0.5% CHAPS, 50 mM DTT, and 24 mM Spermine) or luciferase lysis buffer. Between 35 and 150 μg of extract was mixed with 6x sample buffer and loaded on SDS-PAGE gels. The gels were wet-transferred onto Protran nitrocellulose membrane and the resulting blots were blocked and incubated first in primary antibody and then with the appropriate secondary horseradish peroxidase-conjugated antibody (Dako). The blot was exposed to hyperfilm following the use of enhanced chemiluminescence substrate solution (Amersham Life Science). Primary antibodies were from: Abcam Inc., Cambridge, MA, USA (β-actin, β-Tubulin), Upstate Millipore Corporate Headquarters, Billerica, MA USA (RAS Clone 10), Cell Signaling Technology, Inc., Danvers, MA, USA (Phospho-ERK/MAPK, ERK/MAPK). The V5 epitope is recognized by the mouse monoclonal anti-V5 antibody (Invitrogen, Paisley, UK) and the haemagglutinin epitope is recognized by the anti-HA antibody (mAb) (Covance, Princeton, NJ USA). To detect RAS by immunoprecipitation the rat monoclonal antibody 238 was used (Santa Cruz, CA USA). The mouse and rabbit antibodies to ASPP2 were described previously [9]: rabbit anti-ASPP2 polyclonal antibody ASPP2/77 and mouse monoclonal anti-ASPP2 antibody DX54.10. The mouse monoclonal DO-1 is specific to p53. Signals were detected using ECL detection system (GE Healthcare, Pollards Wood, Chalfont, Buckinghamshire, UK).

### Immunofluorescence

 Cells were fixed in 4% PBS-paraformaldeyde for 15 min, incubated in 0.2% Triton-X-100 for 5 min, then in 0.2% Fish Skin Gelatine in PBS for 10 min and stained for 1 hr with anti-V5 (Invitrogen, Paisley, UK) or with anti-ASPP2 with DX54.10. Antibodies were used at 1:100 dilution in 0.2% Fish Skin Gelatine-PBS, respectively. Staining with the secondary antibody and Hoechst was performed as described [2], followed by visualisation under a fluorescence microscope.

### Immunoprecipitation

 For immunoprecipitation, cells were lysed in IP buffer (20 mM Trish-HCl [pH 7.5], 1 mM EDTA, 1 M KCl, 5 mM MgCl_2 ,_ 10% v/v glycerol, 1% v/v Triton X-100, 0.05% v/v 2-Mercaptoethanol and protease and phosphatase inhibitors). 1-4 mg of lysate was pre-cleared with protein G beads for 30 min at 4°C and subsequently incubated with antibody pre-bound to protein G beads for 2-16 hrs at 4°C. The beads were washed three times with NP40 buffer. The immunoprecipitation beads were mixed with 6x sample buffer and loaded on SDS-PAGE gels. 

### In vitro phosphorylation assay

 2 μg of GST-ASPP2 (693-1128) expressed in *E. coli*, 2 μg of 1mg/ml recombinant Histone 2B or 2 μl of water was incubated in 30 μl of reaction mixture containing 50 mM Tris-HCl, pH 7.5, 0.1% 2-mercaptoethanol, 1 μM microcystine and 100 μM [γ-^32^P]ATP (10 000 c.p.m./pmol) at 30°C for 30 min. 0.35 units MAPK1 or 0.1 units p38 SAPK were used. The reactions were terminated as described [12] and analyzed by chromatography on a C_18_ column or resolved on SDS-PAGE gels and visualized by autoradiograph.

### Quantitative reverse transcription–PCR

Real-time reverse transcriptase–PCR was performed using gene-specific primers (QuantiTect Primer Assays) for human ASPP2 or GAPDH with the QuantiTect SYBR Green RT-PCR Kit (Qiagen, Hilden, Germany). Relative transcript levels of ASPP2 were normalized to GAPDH mRNA levels.

### Statistical analysis

SPSS for Windows (SPSS Inc.) was used to analyze the data. A two-tailed unpaired t-test was used to compare the statistical significance of the differences in data from the two groups.

## Supporting Information

Figure S1 **p38 SAPK is not an efficient kinase for ASPP2 phosphorylation.** ASPP2 (693-1128) fragment was used as a substrate for an in vitro phosphorylation assay by the kinase p38 SAPK. As a negative control no substrate was used and Histone 2B was the substrate for the positive control. ^32^P-labelled ATP was added to the kinase assay and the labelled proteins were resolved on SDS-PAGE gels and visualized by autoradiograph.
(TIF)

Figure S2 **Phosphorylation of ASPP2 by the Raf/MAPK pathway enhances p53-mediated transactivation.** Saos2 cells were transfected with a Bax-luciferase reporter, ASPP2 and p53 and treated with 100 μM PD 98059 or DMSO for 20 hours. The cells were harvested, luciferase activity shown.
(TIF)

Figure S3 **Oncogenic RAS stabilizes ASPP2.** (**A**) Quantitative RT-PCR analysis of ASPP2 mRNA levels in HKe3 ER:HRASV12 cells with indicated treatment. (**B**) HKe3 ER:HRASV12 cells were transfected with ASPP2 wild-type (wt) expression plasmid in the presence or absence of 4-OHT. 16 hours after transfection, 10 μg/ml cycloheximide (CHX) was added to the cells for the time indicated. The protein levels of V5-ASPP2 were determined by western blot analysis. β-Tubulin was used as a loading control. (**C**) Saos2 cells were transfected with ASPP2 wt expression plasmid in the presence or absence of co-transfected HRAS V12. 16 hours after transfection, 50 μg/ml CHX was added to the cells for the time indicated. The protein levels of V5-ASPP2 or HRAS V12 were determined by western blot analysis. β-actin was used as a loading control.
(TIF)
