# Supplementary figures and images for: Phosphorylation of ASPP2 by RAS/MAPK Pathway Is Critical for Its Full Pro-Apoptotic Function
Source: PLoS One. 2013 Dec 2;8(12):e82022. doi: 10.1371/journal.pone.0082022 (PMC3847091; doi:10.1371/journal.pone.0082022)

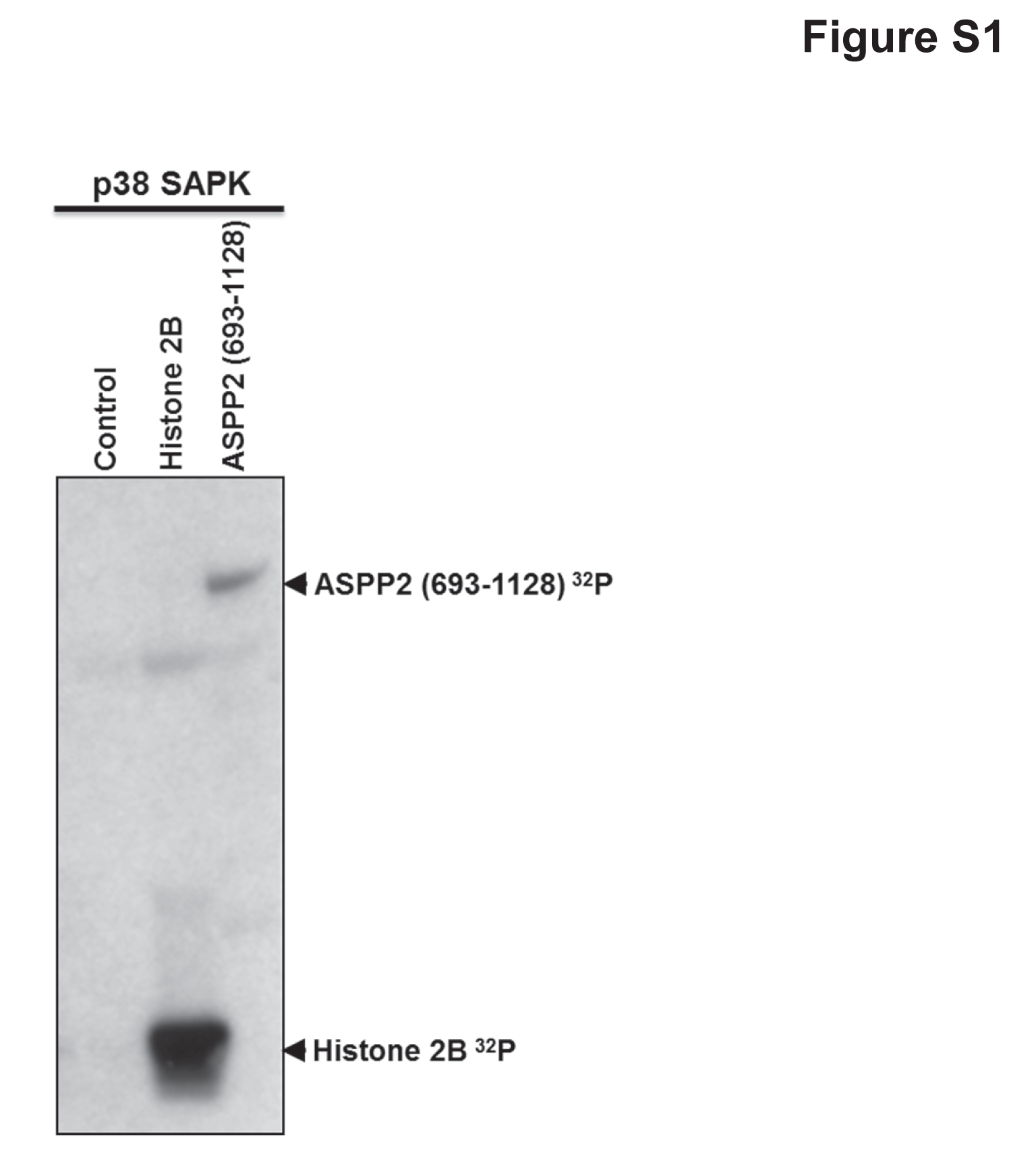

Supplement: Figure S1 — p38 SAPK is not an efficient kinase for ASPP2 phosphorylation. ASPP2 (693-1128) fragment was used as a substrate for an in vitro phosphorylation assay by the kinase p38 SAPK. As a negative control no substrate was used and Histone 2B was the substrate for the positive control. 32P-labelled ATP was added to the kinase assay and the labelled proteins were resolved on SDS-PAGE gels and visualized by autoradiograph. (TIF) [file pone.0082022.s001.tif]

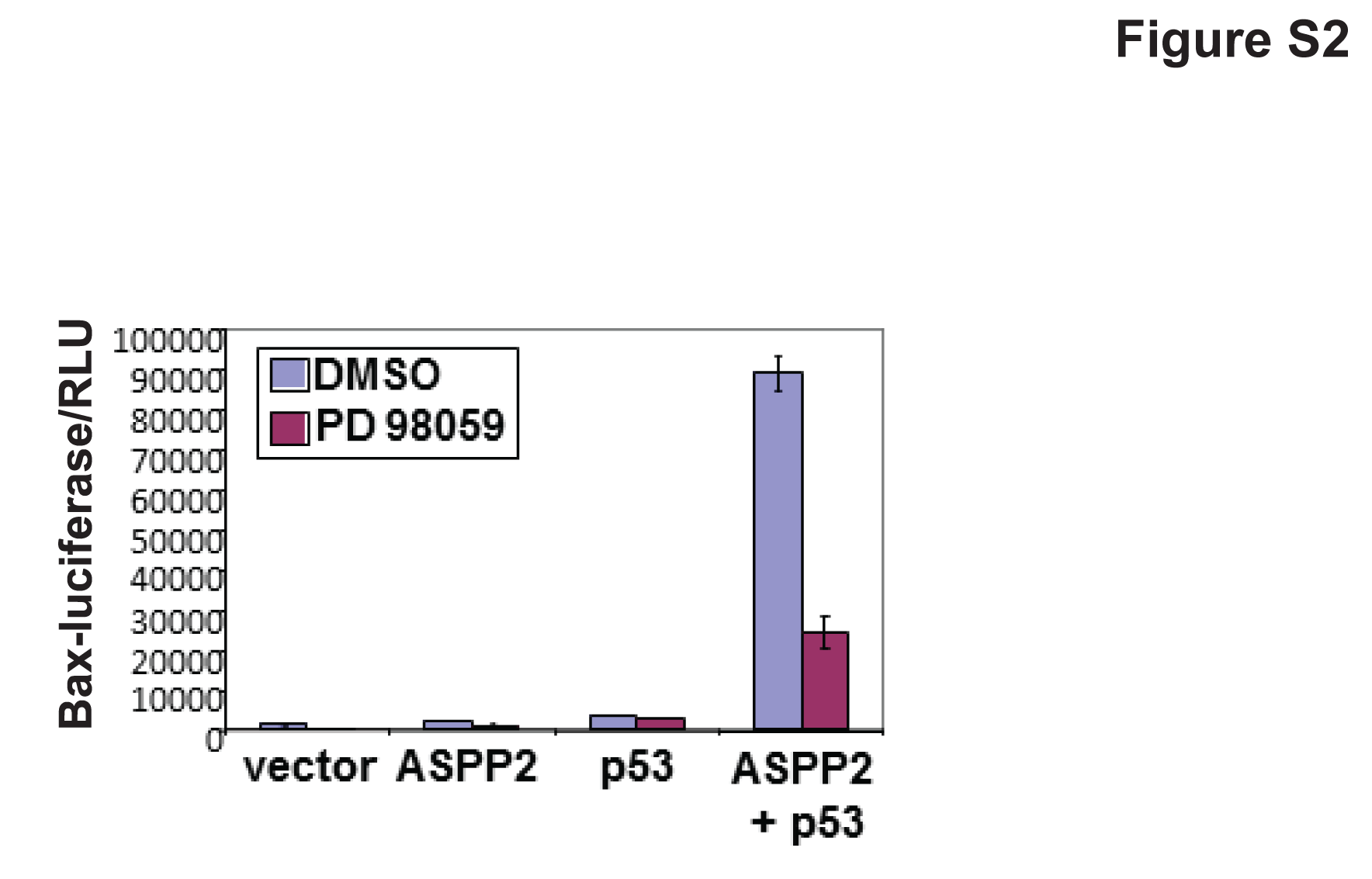

Supplement: Figure S2 — Phosphorylation of ASPP2 by the Raf/MAPK pathway enhances p53-mediated transactivation. Saos2 cells were transfected with a Bax-luciferase reporter, ASPP2 and p53 and treated with 100 μM PD 98059 or DMSO for 20 hours. The cells were harvested, luciferase activity shown. (TIF) [file pone.0082022.s002.tif]

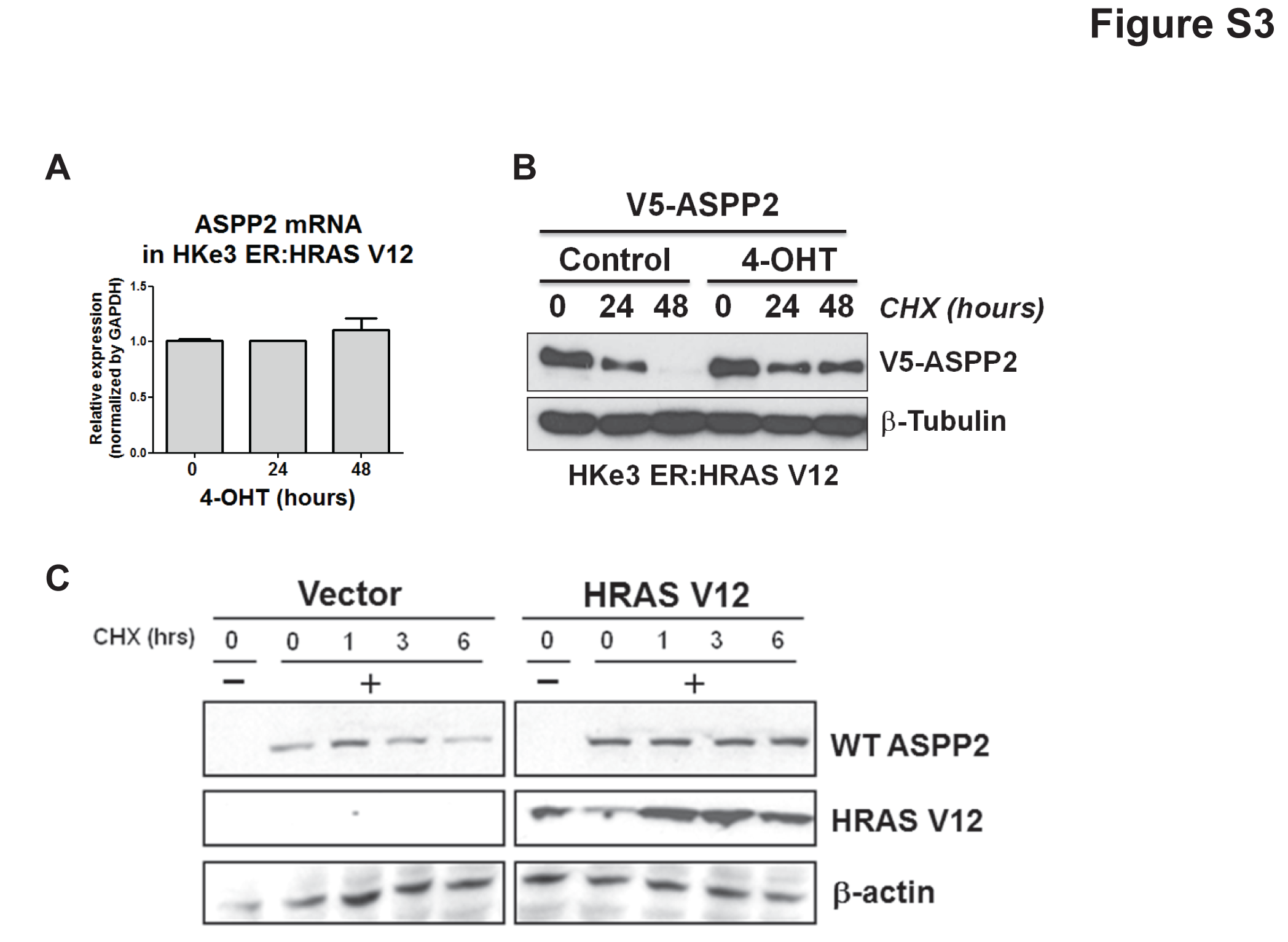

Supplement: Figure S3 — Oncogenic RAS stabilizes ASPP2. (A) Quantitative RT-PCR analysis of ASPP2 mRNA levels in HKe3 ER:HRASV12 cells with indicated treatment. (B) HKe3 ER:HRASV12 cells were transfected with ASPP2 wild-type (wt) expression plasmid in the presence or absence of 4-OHT. 16 hours after transfection, 10 μg/ml cycloheximide (CHX) was added to the cells for the time indicated. The protein levels of V5-ASPP2 were determined by western blot analysis. β-Tubulin was used as a loading control. (C) Saos2 cells were transfected with ASPP2 wt expression plasmid in the presence or absence of co-transfected HRAS V12. 16 hours after transfection, 50 μg/ml CHX was added to the cells for the time indicated. The protein levels of V5-ASPP2 or HRAS V12 were determined by western blot analysis. β-actin was used as a loading control. (TIF) [file pone.0082022.s003.tif]
